# AI Psychometrics: Assessing the Psychological Profiles of Large Language Models Through Psychometric Inventories

**DOI:** 10.1177/17456916231214460

**Published:** 2024-01-02

**Authors:** Max Pellert, Clemens M. Lechner, Claudia Wagner, Beatrice Rammstedt, Markus Strohmaier

**Affiliations:** 1 Business School, University of Mannheim; 2 GESIS–Leibniz Institute for the Social Sciences; 3 Department of Society, Technology and Human Factors, RWTH Aachen University; 4 Complexity Science Hub Vienna, Vienna, Austria

**Keywords:** artificial intelligence, psychometrics, large language model, natural language processing, natural language inference, personality, values, moral foundations, gender/sex diversity beliefs

## Abstract

We illustrate how standard psychometric inventories originally designed for assessing noncognitive human traits can be repurposed as diagnostic tools to evaluate analogous traits in large language models (LLMs). We start from the assumption that LLMs, inadvertently yet inevitably, acquire psychological traits (metaphorically speaking) from the vast text corpora on which they are trained. Such corpora contain sediments of the personalities, values, beliefs, and biases of the countless human authors of these texts, which LLMs learn through a complex training process. The traits that LLMs acquire in such a way can potentially influence their behavior, that is, their outputs in downstream tasks and applications in which they are employed, which in turn may have real-world consequences for individuals and social groups. By eliciting LLMs’ responses to language-based psychometric inventories, we can bring their traits to light. Psychometric profiling enables researchers to study and compare LLMs in terms of noncognitive characteristics, thereby providing a window into the personalities, values, beliefs, and biases these models exhibit (or mimic). We discuss the history of similar ideas and outline possible psychometric approaches for LLMs. We demonstrate one promising approach, zero-shot classification, for several LLMs and psychometric inventories. We conclude by highlighting open challenges and future avenues of research for AI Psychometrics.

In recent years, large language models (LLMs) have been processing an ever-increasing amount of human-generated data. Neural models of language such as GloVe (Pennington et al., 2014), BERT (Devlin et al., 2019), GPT-2 (Radford et al., 2018), XLNet (Yang et al., 2019), RoBERTa (Y. Liu et al., 2019), BART (Lewis et al., 2019), or ChatGPT and GPT-3 (Brown et al., 2020) have come to play a transformative role in several applications of societal relevance. Various authors have referred to these models as “foundation models” (Bommasani et al., 2021; Ribeiro et al., 2020), highlighting that they provide a general-purpose foundation on which future computational systems will be built that can be fine-tuned and adapted for many different application domains and tasks. Examples of such applications include automatically processing millions of résumés in recruiting processes (Kulkarni & Che, 2019), detecting toxic content in social media (Fortuna & Nunes, 2018), identifying fake news and misinformation (Dale, 2017), and creating chatbots for text-based human–computer interaction (Adiwardana et al., 2020). The increasing reliance on such artificial intelligence (AI) tools has also raised important concerns. One of these concerns is that LLMs, because they were trained on human-produced texts, may contain a variety of built-in biases, such as racial bias, gender bias, or extremist views. Such biases and views may manifest in the models’ behavior (e.g., the text they generate), which in turn may adversely impact individuals and social groups when models are used for screening applicants during recruiting or admission processes, monitoring social media posts, powering chatbots and virtual assistants, or other applications.

But how can the potential biases and views ingrained in LLMs, and their characteristics more generally, be detected and ideally quantified in a principled fashion? A common way of identifying biases or, more generally, views (e.g., values, attitudes) held by humans is to conduct psychological assessments (Fiske & Pearson, 1970; Watson, 1932; West & Finch, 1997). Traditionally, psychological assessments of humans have been the domain of psychometrics, a subdiscipline of psychology that concerns itself with the science of psychological measurement (Furr & Bacharach, 2014; Nunnally, 1994; Rust & Golombok, 2014). The main focus of psychometrics, at its inception, has been the measurement of cognitive abilities (“intelligence”), an area that inspired the development of fundamental measurement theories, such as classical test theory or item response theory, and has resulted in a large number of standardized cognitive tests that are in wide use today. However, crucially for our present article, work in psychometrics over the past decades has produced a wider array of well-validated tests that enable the assessment of “noncognitive” constructs, such as personality traits, values, or attitudes. Although many different assessment formats exist, most of the assessments in this domain are language based. That is, they consist of a series of items (i.e., questions or statements) that respondents answer by giving a rating on a standard response scale with verbal and/or numeric labels. We will summarily refer to such multi-item surveys of psychological characteristics as “inventories” in the remainder of this article.

On the basis of these observations, we argue that psychometric inventories—similar to the way they are used to assess humans—can be used as diagnostic tools that provide a window into the “psychological” characteristics, metaphorically speaking, of LLMs. Although we by no means aim to anthropomorphize artificial intelligence, we argue that LLMs can exhibit—or, more precisely, mimic—the very same psychological characteristics that are typically studied in humans. This is due to LLMs being trained on vast corpora of human-written text that routinely contain statements related to human values, attitudes, beliefs, and personality traits. Such models will inadvertently but inevitably acquire (“learn”) a set of psychological characteristics during the training process. These learned characteristics will ultimately give a unique psychological profile to every such model that may differ from other models, not unlike the individual differences observed in humans. Akin to how the values, attitudes, and personality traits of humans become manifest in their behavior (broadly conceived), the psychological profiles of an LLM may in turn manifest in the model’s “behavior.” In this context, speaking metaphorically, “behavior” means the models’ outputs in the wide variety of downstream tasks for which they may be used. Accordingly, we submit that it should be possible to assess these psychological characteristics in LLMs through psychometric inventories (i.e., language-based assessments) originally developed for humans.

In a series of demonstrations, we provide various LLMs with questionnaire items from different inventories as input and “ask” the models to choose an answer on the verbal rating scale as its output. The models’ responses open a lens through which to explore potential biases ingrained in LLMs in a principled, information-rich, and scalable way. This approach of studying the characteristics of LLMs through psychometric inventories may ultimately help to avoid the development of LLMs that induce harm when deployed in broader societal applications. We conclude our article by arguing that our investigations give new impetus to the interdisciplinary field of research that we would refer to as “AI psychometrics.” We propose that AI psychometrics should focus on tackling the manifold research opportunities and challenges that emerge when deploying psychometric inventories to LLMs.

## A Very Brief History of Psychometrics and AI

The idea of applying psychometric assessments to AI was already discussed in the first decades after AI’s foundational period in the 1950s (Bringsjord, 2011). Pioneering work by Thomas G. Evans described a computer program that could solve a subtask of geometrical analogy reasoning that was part of an intelligence test battery from the 1940s (Evans, 1964). The idea was that a program that could eventually compete with humans in some part of actual tests of human intelligence could be considered intelligent too. This early attempt of linking AI and psychometrics fell within the rather narrow bounds of the then-current “good old-fashioned artificial intelligence” (GOFAI) paradigm with the goal “to build useful computer systems, doing, or assisting with, tasks that humans want done” (Boden, 2014, p. 89). Similar approaches were also proposed by other foundational figures of AI, such as Allen Newell. Newell (1973) described the need to consolidate the disparate experimental results in (cognitive) psychology of his times into one body of knowledge in AI to progress. Among the three possible ways to achieve this that Newell outlined, the approach he apparently preferred was psychometric: “A . . . mold for such a task is to construct a single program that would take a standard intelligence test, say the WAIS [Wechsler Adult Intelligence Scale] or the Stanford-Binet” (Newell, 1973, p. 305).

However, despite their merits, these early attempts conceived psychometrics mainly in terms of cognitive tests and intelligence with a focus on performance assessment. This was fully in line with the general focus in the field of AI on cognitive tasks such as planning and problem-solving at the time. A popular early criticism then concerned the inability of AI to operate outside this realm of “cold cognition,” that is, displaying “inhumane” intelligence with no emotional basis and lacking the motivational complexity of thought (Neisser, 1963). In response to such criticism, Herbert Simon (1963) showed that “hot cognition” concepts, such as emotional behavior, could be integrated in the supposedly cold models. Of course, fully understandably because of the types of models available at the time, the integration of such hot cognition concepts remained at a very basic level of introducing emotions as “interrupt systems” affecting program control, changing the goals to orientate to and introducing responses.

In the early 2000s, “psychometric AI” was discussed explicitly as providing an answer to the old question, “What is AI?”:

Psychometric Al is the field devoted to building information-processing entities capable of at least solid performance on all established, validated tests of intelligence and mental ability, a class of tests that includes not just the rather restrictive IQ tests, but also tests of artistic and literary creativity, mechanical ability, and so on. (Bringsjord & Schimanski, 2003, p. 889)

## The Rise of Large Language Models

AI has evolved dramatically in the 60 years that have passed since the first attempts of linking psychometrics to AI that focused on cognitive tasks. Even when compared with the early 2000s, progress has been remarkable, indicating a qualitative shift in the capabilities of AI. To understand this change, it is important to note that the field of natural language processing has undergone a radical transformation around the years from 2017 to 2018 with the advent of transformer architectures as an integral part of novel LLMs. Whereas it is an open discussion whether those emerging new architectures really began to “understand” language better (Mitchell & Krakauer, 2023), they nonetheless showed drastically increased performance on a wide variety of traditional and novel tasks, such as automated translation of text, text summarization, and detection of textual entailment. Various metrics, such as precision, recall, or BLEU scores (Papineni et al., 2001) computed using “benchmark” test suites consisting of different language-related subtasks on canonical data sets, provide evidence of that progress. Although such approaches can naturally be concerned only with some more or less isolated aspects of natural language and may therefore appear fragmented or stylized, we should refrain from downplaying advances that have been made (S. Bowman, 2022) and from making unfair comparisons (Firestone, 2020). In fact, recent model developments have advanced the state of the art of natural language understanding and generation so significantly that human-like performance was reached on benchmarks, such as GLUE (Wang et al., 2018), that include tasks judging English acceptability or establishing whether pairs of questions are semantically equivalent. This led to the development of supposedly much harder benchmarks, such as SuperGLUE (Wang et al., 2020), that have been broken quickly nonetheless.

These recent developments highlight that LLMs have reached a stage at which they can reach human-like performance on many different tasks assessing language understanding and generation capabilities. Crucially, these enhanced capabilities of LLMs also comprise the ability to engage in hot cognition and to exhibit (or, more precisely, mimic) human-like characteristics and behaviors beyond the purely cognitive realm. Although the traditional focus of testing AI on cognitive tasks has also resurfaced in recent approaches to subject LLMs to standard cognitive tests (e.g., Binz & Schulz, 2022), LLMs are capable of much more than the cold cognition required by these cognitive tests. Simple affective mechanisms do not have to be explicitly introduced into the model architectures in the way in which early proponents such as Herbert Simon envisioned. Instead, models trained on large amounts of text in a self-supervised way can exhibit rich psychological traits that so far have been studied only in the human realm. Potentially, such noncognitive traits could become important if LLMs and solutions built on them are going to be employed in contexts in which they perform tasks and make decisions that have real-world consequences for individuals and social groups. Their traits, inadvertently but inevitably acquired during the models’ training with text generated by humans, are likely to influence the “behavior” (i.e., output) of these very models.

## Opportunities for Psychometric Assessment

The unprecedented capabilities of LLMs open up an opportunity for a more inclusive approach to AI psychometrics, one that spans the full spectrum of socially relevant traits, including noncognitive traits such as personality, values, morality, and attitudes. We argue for the need of further empirical studies of not only the cognitive (Binz & Schulz, 2022) but also these noncognitive characteristics of LLMs and to establish how they relate to behavior and decisions of LLMs in downstream tasks, a goal that is echoed in the related research program of “machine behavior” (Rahwan et al., 2019). As we demonstrate, with language and text as the shared foundation of both psychometric inventories and LLMs, we can leverage existing survey instruments to learn about the hidden values, attitudes, and beliefs that are encoded in these models. Such research will result in a more complete understanding of the characteristics and potential biases built into these foundation models (Simon, 2019). In the present article, we aim to pave the way for such an expanded approach to AI psychometrics.

We see several possible approaches to assess the psychological profiles of LLMs through psychometric inventories. These approaches differ mainly in how they elicit the models’ responses to the questionnaire items. We describe these approaches as masked-language prediction, next-word prediction, and zero-shot classification. Although all approaches are viable in principle, in the section Methods for Model Assessments in the appendix, we explain why our focus will subsequently be on zero-shot classification for our empirical demonstrations.

## Demonstrations of AI Psychometrics

**Fig. 1.**
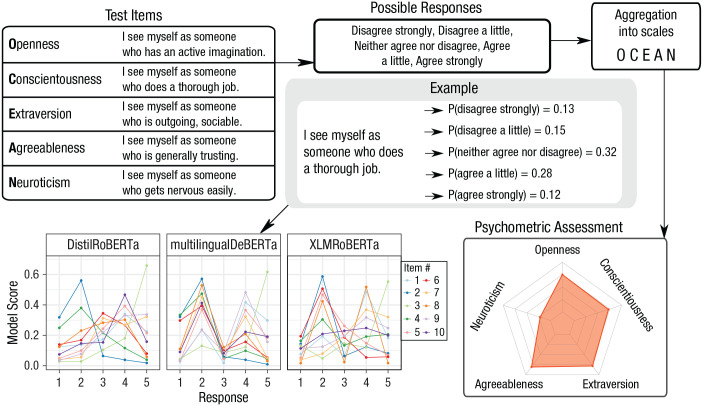
Illustration: How psychometric assessments could be adapted to large language models. Taking items and responses from the Big Five Inventory (BFI) as examples, we show the steps of one possible assessment scheme. We present the model one by one with each of the survey items and the possible responses. We retrieve the model’s distribution of probability scores over responses (panel “Example”). Scores are aggregated into scales that can be visualized and used for further analyses. This figure illustrates the workflow of one possible way to psychometrically assess large language models.

Figure 1 illustrates the zero-shot classification approach we will use in the subsequent demonstrations using a widely used personality inventory as a case in point: the 44-item Big Five Inventory (BFI), available in English and German (John et al., 2008; Rammstedt, 1997). As (nonhuman) respondents, several LLM architectures were chosen, shown in Table A1, that follow the blueprint laid out by the original BERT model class (Devlin et al., 2019), such as RoBERTa (Y. Liu et al., 2019) and DeBERTa (He et al., 2021). We do not include GPT-3, GPT-4, or ChatGPT in our present demonstrations because, as generative models, they are prone to the issues that we describe in the subsubsection Next-Word Prediction, such as stochastic outputs and sensitivity to the order of input examples. As explained before, these issues are circumvented by our proposed assessment scenario using a natural language inference (NLI) approach that we use to analyze a diverse set of open-sourced models. Another reason to exclude GPT-3 (and similar models with limited access only) is the lack of transparency arising from the fact that the model is not fully, locally accessible to us. Sanitization of outputs may happen behind the scenes without documentation, making it hard for researchers to know which version of the model they are analyzing. In the interest of open science, with our approach we are able to provide materials (see the repository linked in Open Practices) that allow the scientific community to fully replicate our analyses on the exact same models that we used without having to pay for access to potentially different models available only through an application programming interface (API) or a web interface. We do not analyze any of the GPT models directly, but we do include the openly available BART model (Lewis et al., 2019) that incorporates elements of both GPT and BERT architectures.

For a detailed description of the process used to elicit responses to questionnaire items from the models, see the section Example of Our Approach in the appendix.

### Assessing personality

What kind of “personalities” do the AI models have? Do they exhibit a socially desirable profile—or do they possess characteristics that are commonly viewed as undesirable or even problematic? To approach this question, we first assess global personality in terms of the Big Five personality dimensions by use of the BFI (John et al., 2008). The BFI assesses the Big Five dimensions (openness, conscientiousness, extraversion, agreeableness, and neuroticism) with 44 items that are each to be rated on a 5-point scale ranging from *disagree strongly* to *agree strongly*.

Additionally, we assessed undesirable and offensive (though not necessarily pathological and relatively widespread in human populations) personality traits delineated by the dark tetrad. The dark tetrad consists of the traits Machiavellianism (i.e., manipulative interpersonal behaviors), narcissism (i.e., excessive self-love), psychopathy (i.e., lack of empathy), and sadism (i.e., intrinsic pleasure in hurting others). We employ the Short Dark Tetrad questionnaire (Paulhus et al., 2021), which assesses these four traits with 28 items (seven per trait) that are answered on a 5-point scale ranging from *disagree strongly* to *agree strongly*.

**Fig. 2.**
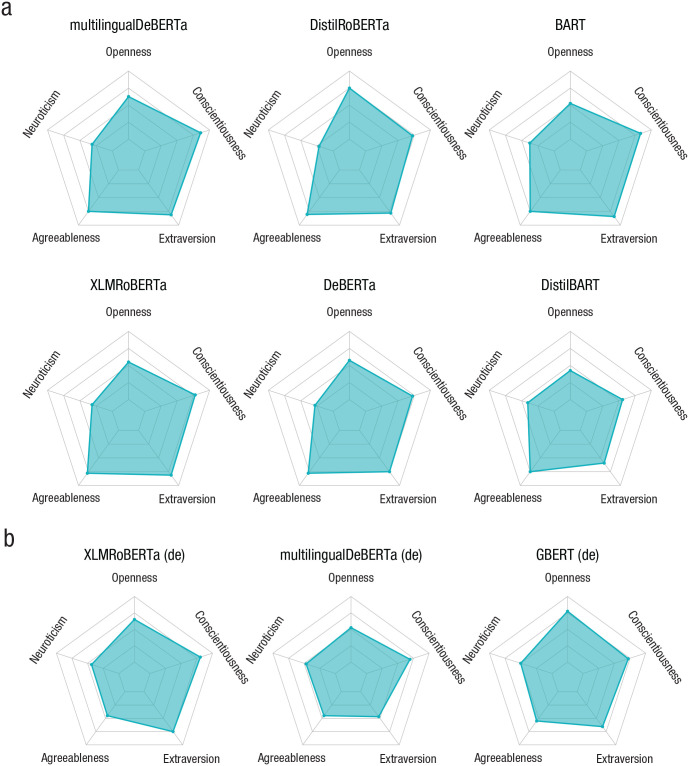
Assessing personality via the 44-item version of the Big Five Inventory for different models. Panel A shows the English and panel B the translated German version version of the questionnaire. Using multilingual models enables us to study cross-lingual (and potentially cross-cultural) differences of model scores and survey items. Model results generally show no surprising outliers and overall balanced personality profiles.

Results for the Big Five are displayed in Figure 2 for the English (panel A) and for the German language models (panel B). The emerging personality profiles for the six English language models are surprisingly homogeneous. All models score more or less equally high on agreeableness and extraversion and low on neuroticism. Slight differences are observable for conscientiousness and especially openness. Whereas the second model (DistilRoBERTa) scores higher on openness, the last model (DistilBART) reports comparatively low scores on conscientiousness. Within the three models using the German version of the BFI44 (including an additional item for agreeableness; Rammstedt, 1997), we see more pronounced differences among the models: Whereas XLMRoBERTa’s and GBERT’s profiles are high on openness, extraversion, and conscientiousness, DeBERTa scores high only on conscientiousness. Interesting is the comparison within models across languages. Whereas the English version of XLMRoBERTa scores higher on agreeableness compared with its German counterpart, for multilingualDeBERTa, the English version also scores higher on agreeableness but also on openness and extraversion than the German version. Such a comparison—especially if also conducted on the level of single items—can also be useful from a methodological point of view: Systematic differences across language versions could be seen as a first indication for biases caused by the translation of items or by systematic differences in the model-training data between languages.

**Fig. A1.**
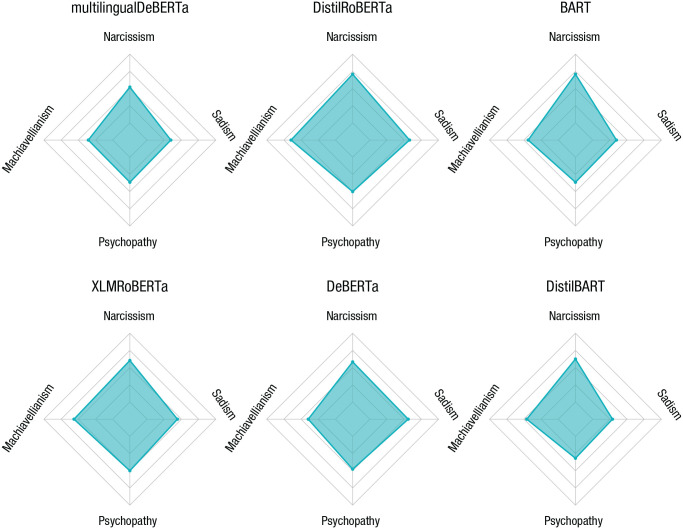
Assessing personality via dark tetrad. Unusually accentuated scores on this assessment may reveal potential for conflicts with no-harm objectives in model decisions. Our assessment shows that the models lie well within the range of values observed in standard human populations, not displaying worrying pathological features.

Overall, the Big Five profiles appear characteristic of a relatively balanced and well-adapted personality (low neuroticism; high conscientiousness, agreeableness, and extraversion). They yield little indication that any of the models possess an extreme, accentuated personality. A more direct assessment of a potential “dark side” of these models’ personalities, however, is offered by the models’ scores on the dark tetrad. These results are shown in Figure A1 in the appendix. Again, the results do not suggest unexpected personality profiles. Most models score low (between 2 and 3 on the 5-point-scale) on all four dark traits. Only a few exceptions stand out, such as the high narcissism scores of DistilRoBERTa and BART. Overall, the models we study here generally do not score highly on socially undesirable, potentially problematic traits. Contrariwise, they score well within the range observed in normal human populations, where the dark traits are roughly normally distributed around the scale’s midpoint (Paulhus et al., 2021).

### Assessing value orientations

Next to personality traits, value orientations are another central aspect of a person’s psychological makeup. Values are beliefs about desirable end states or modes of conduct that vary in importance, transcend specific situations, and guide the selection or evaluation of behavior, people, and events (Schwartz, 1992). Whereas Big Five traits describe dispositional behavioral tendencies (i.e., how the person typically acts), value orientations describe dispositional evaluative tendencies (i.e., what the person cherishes or finds important in life). The most prominent and best-validated (including cross-culturally) model of human values is Schwartz’s (1992) theory of basic human values. This theory distinguishes 10 basic human values (or, in its refined version, 19; Schwartz et al., 2012) that emerge with great regularity in samples of human respondents from across the globe. We used the recent 57-item Revised Portrait Values Questionnaire (PVQ-RR; Schwartz & Cieciuch, 2022) to assess these basic values. This inventory relies on a portrait format in which each item consists of a statement describing a person in terms of their values (e.g., “Thinking up new ideas and being creative is important to her”). Respondents indicate how similar they are to the person described in the statement on a 6-point scale ranging from 1 = *not like me at all* to 6 = *very much like me*.

Using this inventory opened a window into the values espoused by the six English language models. Because the PVQ-RR comes in a male version (containing the pronouns “he” and “him”) and a female version (containing the pronouns “she” and “her”), it also enables us to establish differences in the models’ scoring across the two gender versions. Differences in what response the models think is entailed by each item depending on what gender pronouns the questionnaire uses can be taken to indicate gender bias in these models.

**Fig. 3.**
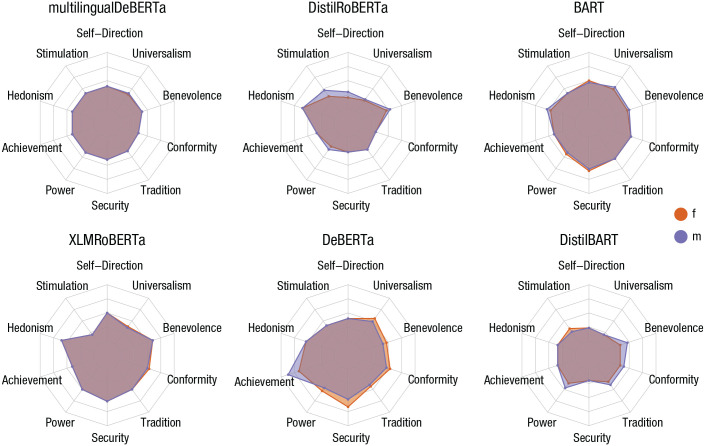
Assessing value orientation via the revised Portrait Value Questionnaire (PVQ-RR). Radar charts show results for the questionnaire version with male (pastel blue) and female pronouns (reddish orange) with otherwise identical items. Purple-gray areas correspond to agreement between the two gendered versions. The slight differences visible (areas in either one of the two colors) point to the existence of gender biases of the models.

Figure 3 shows results for the 10 values. From the visual pattern, one can immediately see that most models score low on most dimensions, meaning that they assigned higher probabilities to the lower ends of the response scale. For multilingualDeBERTa and to some extent BART, we observe little differentiation between the 10 values. The other models show more differentiation, attaching a lower importance to some values and higher importance to others. For example, DistilROBERTa scores relatively low in all values except hedonism and stimulation as well as, to some extent, benevolence. DeBERTa scores high in achievement.

There are indeed some indications for built-in gender bias for some of the models, although the score differences for the two gender versions of PVQ-RR mostly appear small. The largest difference we observe is the “male” achievement score of DeBERTa, which is noticeably higher than the “female” score on the same value.

### Assessing moral norms

Can LLMs also reflect moral beliefs and norms that they absorbed from text during training? Various studies have already explored this question. For example, early work on this subject computed distances of vector representations between statements and moral concepts on an atomic level (Jentzsch et al., 2019). The proliferation of more capable language models has enabled more sophisticated ways of exploring this issue, such as directly asking the models moral questions (“Should I kill people?” “Is it allowed to murder people?” with simple answer templates of “Yes [no], I should [not]”; Schramowski et al., 2019, 2022). Compared with our approach, which uses established psychometric inventories, this more ad hoc approach is somewhat less standardized and systematic.

**Fig. 4.**
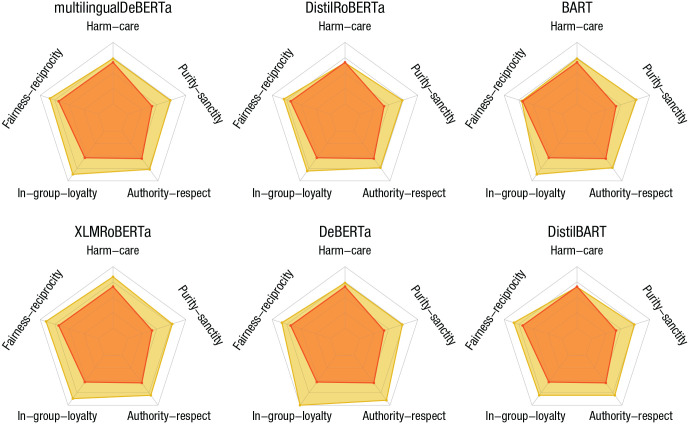
Assessing moral norms via the Moral Foundations Questionnaire. Models tend to deviate from the average, politically moderate American’s scores (red) as reported by the developers of the questionnaire (Graham et al., 2011). The models (yellow) usually deviate in the direction of putting more emphasis on those moral foundations that are associated with conservative political orientations.

In our demonstration, we illustrate the direct application of the established Moral Foundations Questionnaire (Graham et al., 2009, 2013; Haidt, 2007) to various models. Figure 4 contrasts reported moral beliefs from average, politically moderate Americans (Graham et al., 2011) with model scores. Across different models, we can observe that models put stronger emphasis on moral norms such as authority-respect, in-group-loyalty, and purity-sanctity than the human reference group did. Interestingly, the moral norms that are stressed more by the models are usually associated with individuals holding conservative political views. This suggests that there might be significant differences across various dimensions between the moral beliefs held by people and the moral beliefs absorbed by language models from large corpora. Corroborating or refuting such initial observations in future endeavors should be an important and critical concern for researchers aiming to design responsible AI systems.

### Assessing beliefs about gender

Previous research found gender bias in algorithmically curated online environments (Vlasceanu & Amodio, 2022) and specifically also in LLMs (Caliskan et al., 2017), which indicates that there is a need for monitoring such encoded gender and sex diversity beliefs. Typically, researchers assume the gender-binary framework, which suggests that humans comprise only two types of beings, men and women. This framework has been challenged by both academic research and social activism (Hyde et al., 2019). To address this issue, social scientists have developed novel instruments to measure beliefs about the ontology of gender and sex. Assessing those beliefs is important because prejudice against or affirmation of gender and sex minorities (i.e., transgender, nonbinary, and gender or sex diverse) is often framed in terms of beliefs about the ontology of gender and sex or about gender and sex diversity.

We use the recently developed Gender/Sex Diversity Beliefs Scale (GSDB; Schudson & van Anders, 2022) to measure the biases and prejudices against sex and gender minorities that are encoded in LLMs. The scale consists of five factors. Items that recognize the existence of gender and sex diversity loaded positively on *affirmation*. Those associated with denying gender and sex diversity loaded negatively. *Gender normativity* is composed of items about the importance of femininity for women and masculinity for men and the inauthenticity of non-normative gender expressions (e.g., femininity among men). *Uniformity* contains items that stress that people of the same gender or sex are similar to each other. Items that describe genital surgery as a necessary precondition for a person to “truly” transition genders or sexes load on *surgery. Upbringing* collects items about the role of upbringing and early experiences in determining gender or sex.

**Fig. 5.**
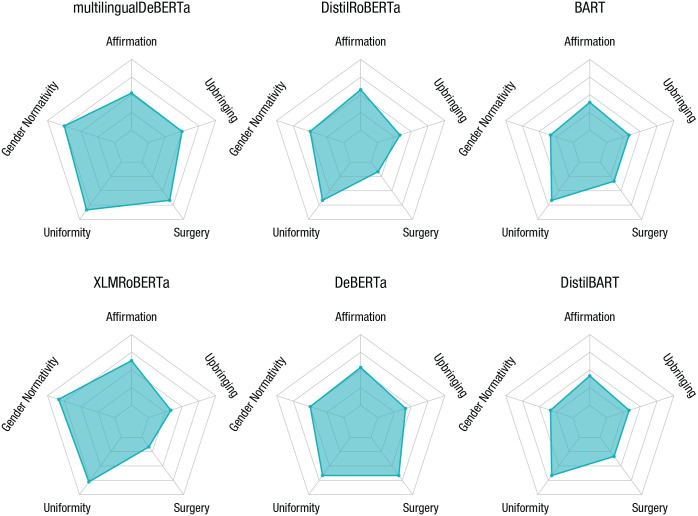
Assessing beliefs about gender via the Gender/Sex Diversity Beliefs Scale (GSDB). The models display uniform views of people of the same gender or sex and little affirmation of diversity. This points to potential issues of the models to take nontraditional aspects of gender and sex adequately into account.

All factors except upbringing are associated with feelings toward gender and sex minorities that were either negative (gender normativity, uniformity, surgery) or positive (affirmation). Our results (Fig. 5) show that all language models have two things in common: (a) an emphasis on uniformity (i.e., people of the same sex or gender are similar) and (b) lack of affirmation, that is, they do not reveal strong positive feelings toward gender and sex minorities (e.g., language models tend to disagree with statements such as “There are many different gender identities people can have” or “Nonbinary gender identities are valid”).

## Open Challenges and Conclusions

Our demonstrations highlight the feasibility of using psychometric inventories as a window through which to study the characteristics of LLMs as well as to identify and monitor differences between various models. At the same time, our approach is only one of many different ways in which research in AI psychometrics can be pursued. Without any claim of completeness, we now want to discuss a number of future research challenges that we deem important.

### Reliability and validity of psychometric assessments of AI

We see a wide field of open methodological and ethical questions and challenges related to psychometric assessments of LLMs. A continued effort to probe the validity and reliability of reusing human psychometric assessments in the domain of AI is necessary. As an example, current models have been claimed to display results in theory-of-mind (TOM) tasks that are comparable to the performance of elementary school children (Kosinski, 2023). Problems have quickly been reported with those findings, as small variations that keep TOM principles intact make the results disappear (Ullman, 2023). Another important, general question concerns self-consistency of LLMs. So far, from our demonstrations we can provide only partial answers: We do not provide the model with explicit information about the ranked order of possible responses. However, it seems that the models establish this property implicitly on their own, judging from the example distributions over items shown in the lower part of Figure 1 (the data are available for all models under consideration in our replication materials; see Open Practices). This emerging feature can be be seen as first evidence that the models do not display an obvious lack of self-consistency: They are at least consistent in the sense that there is no obvious bimodality observed in the probability distributions, as, for example, high values for both extremes of the scales—*totally agree* and *totally disagree*—as equally likely would indicate. We want to note here that this issue should definitely be further investigated in future research. Adversarial testing that highlighted such inconsistencies in other domains (Camburu et al., 2020) could help us to make our approach more robust. From a more high-level perspective, we could also use different, but related, questionnaires and compare if model responses follow a similar pattern, for example, for the Genderism and Transphobia Scale (Hill & Willoughby, 2005) and the Gender Role Attitudes Scale (García-Cueto et al., 2015).

### Stability of psychometric profiles

Future research can tackle many interesting and creative research questions, such as the following: Does text scraped from specific parts of the Internet lead to specific characteristics of models trained on that text (e.g., from special communities on Reddit or 4chan)? Do models trained on books or movie plots preferred by certain personality types develop similar traits? Does the accidental filtering of text from and about sexual-minority groups in large pretraining corpora influence the diversity conceptions of LLMs (Dodge et al., 2021)? Research projects that tackle these questions produce insights on an important general question: To what extent do models absorb (psychometric) aspects of their underlying training data? To systematically study the role of data in isolation, we can compare model series featuring the exact same architectures and the exact same training parameters and differing only in regularly updated training corpora (Loureiro et al., 2022).

### Engineering psychometric properties of AI

Assuming that psychometric assessments of LLMs can be done in a valid and robust manner, it may be feasible to deliberately manipulate the personality traits, values, and attitudes that LLMs exhibit. Doing so may help to change the behavior of models in a desired fashion (e.g., reducing bias or dark traits). It may also open up new opportunities for research: Conducting in silico experiments may create a safe experimental space for exploring novel psychological research questions that could not have been addressed previously because of ethical and other concerns. We saw that using language models to simulate subpopulations with certain demographics seems possible (Argyle et al., 2023). Such a synthetic sampling approach can also be used to study phenomena on the level of individuals as in psychometrics. We can potentially apply those quick and cheap methods on the full range from pretests to full and deep investigation of hypotheses.

### Multimodal psychometric assessments of AI

Although we focused here on psychometric assessments of LLMs through language-based inventories, similar ideas are applicable to other modalities. In the visual domain, we can imagine a Rorschach-like test scenario of image creation to investigate, for example, which colors map to which feelings and other abstract concepts for these models, thereby potentially revealing cultural norms. Sound and video generation may offer additional glimpses into inner workings of models. In the future, we may be able to easily combine those ways to assess psychometric properties of more general, multimodal AI systems.

### Lifelong monitoring of psychometric properties of AI

Although we want to refrain from attributing human-like capabilities and traits to AI technologies and to talk about AI in anthropomorphic terms, we want to highlight the need to further develop monitoring tools and test suites to support the lifelong monitoring of AI tools and to shed light on their imperfections, biases, and harmful consequences. For that endeavor, having unrestricted access to local, “frozen” versions of models to do full inspections of them is a necessary condition. Researchers have noticed examples of apparent “sanitization” or other corrections of model text outputs happening behind the scenes to models available only through APIs or web interfaces (Rozado, 2023). In the interest of transparent documentation, it is important to know which exact version of the model we are analyzing. This issue can be expected to play an even bigger role in the future, especially so with the proliferation of approaches that let models directly adapt to humans, for example, by reinforcement learning from human feedback (Ziegler et al., 2020).

### Consequences of psychometric profiles of AI

One current limitation of our demonstrations concerns the question of practical relevance: More empirical research is needed to establish how precisely certain traits of AI models influence their behavior in downstream tasks. These future research endeavors could analyze models that are able to use external tools such as APIs and search engines (Schick et al., 2023). Psychological profiles could potentially be an explanatory factor of a specific choice between those tools that a model has access to. The choice of querying unreliable sources of information, for example, could depend on whether the model displays risk-averse traits or not, especially in nuanced contexts where it is not easy or even possible to give a right or wrong answer. Also in robotics, researchers make use of LLMs (Huang et al., 2022; Li et al., 2022). Here in particular, behavior often involves planning and directly acting in the real world, which makes monitoring potentially harmful model features especially important. Known examples of undesired model behaviors have so far been uncovered using rather simple pipelines that rely on mask filling or text generation using curated templates (Gehman et al., 2020; Liang et al., 2022; Nangia et al., 2020). The proposed text choices by the models are usually judged by criteria such as being hurtful or stereotypical (Abid et al., 2021; Nozza et al., 2021, 2022). The problems arising from the ad hoc quality of many of such tests have been pointed out (Blodgett et al., 2021). As a future complementary perspective, we can enrich those approaches more and more by integrating knowledge from the social sciences in general and from psychometrics specifically. As we have shown, we can adapt existing methods from those domains in a rather straightforward way.

The ongoing (and continuing) trend of language models to underpin ever larger parts of technology will likely make them play a more and more important role in our future daily lives. In our view, the research community should clearly make use of the opportunity to describe various psychological aspects of models via rich psychometric profiles. This offers an exciting and valuable avenue for future research to adapt well-established methods from human psychometrics and study the relationship of such assessments to all kinds of other phenomena (like decision-making or other important behavior of AI). In the future, we may be able to uncover more and more relationships and to offer robust assessments of the real-world consequences of psychometric traits of AI.

### Conclusions

We demonstrated how standard psychometric inventories that were developed to assess “noncognitive” psychological characteristics in humans, such as personality, values, morality, or beliefs, can be repurposed as diagnostic tools to assess analogous characteristics in LLMs. Similar to how human respondents fill in a questionnaire, LLMs respond to questionnaire items by returning a probability of entailment for each verbally labeled response option through zero-shot classification. These responses are then aggregated to scale scores using standard scoring rules of the inventories to obtain the levels of the model on each given trait (e.g., low agreeableness).

In doing so, we built on a rich history of research linking psychometrics and AI, which has mainly focused on cognitive assessments. By contrast, the inventories employed in our demonstration capture noncognitive characteristics that the LLMs inadvertently but inevitably acquire from the vast text corpora on which these models were trained. Sedimented in these texts are the beliefs, values, personalities, and biases of the innumerable and diverse human authors who produced these texts. The way in which the models acquire such traits from texts is complex, opaque, and poorly understood so far; yet it is clear that this learning process is channeled and constrained by the models’ neural architecture and subject to various deliberate and nondeliberate human interventions that may influence these traits (e.g., the selection and curation of the text corpus, purification steps, potential fine-tuning on annotated text). There are some obvious parallels to how humans acquire psychological traits through their ongoing interactions with the social and physical world, channeled and constrained by their nervous system and subject to deliberate and nondeliberate human interventions (e.g., education and discipline).

The analogy to human psychometrics is thus quite far-reaching and intriguing. At the same time, it is important not to overstretch the analogy and to be mindful that the foregoing description is mostly metaphorical. One must not fall into the trap of anthropomorphizing AI models that are mere prediction machines. Different from humans, the traits that LLMs exhibit are purely based on language and thus far more narrow than the rich mental world of humans, which is linked to their complex physiology and embedded in multilayered physical contexts, just as the range of behaviors that these models can perform is quite limited. At the same time, the traits and attendant behaviors of LLMs can still be quite consequential for actual individuals and social groups if the models are deployed in real-world applications such as the ones described at the outset.

It is equally important to realize that several of the aforementioned assumptions are so far untested. As we highlighted, there are many open questions—both conceptual and technical in nature—that have yet to be resolved. Still, we believe that our demonstrations clearly highlight the novel potentials of the interdisciplinary field of research on the intersection of disciplines such as psychology, linguistics, and computer science, which we refer to as “AI psychometrics.” This area offers a wide variety of research questions and several directions to explore that we consider important not only for future research but also because of the far-reaching social and economic implications of AI that are only going to become more pronounced in the coming years.

## Appendix

### Methods for model assessments

#### Masked-language prediction

The first possibility is to make use of masked language modeling (MLM), which is the common way of training large language models in the BERT tradition. To create large language models from scratch (i.e., to pretrain empty model architectures), researchers gather large corpora of text from the Internet (Gao et al., 2020; Ortiz Su’arez et al., 2019, 2020). They then feed those texts to the model, removing (i.e., “masking”) one token (which roughly corresponds to a word) from a sequence of text at a time and asking the model to predict that token. The training objective for the model consists of learning to predict the right token over many iterations. On each iteration, mechanisms are operating to numerically assess how correctly the model chose a possible token and to revise its weights in case it did not choose the correct one from the original, unmasked sequence. This schematic sketch illustrates that the training procedure is so far unsupervised (also called “self-supervised”) because it does not involve any step in which the models receives human-annotated (supervised) training data. This step usually follows later in the so-called fine-tuning phase, in which a pretrained model is presented with data, such as comments from social media labeled by humans for toxicity, to enable the model to learn how to classify these data. For psychometric assessments of large language models, we do not necessarily need to fine-tune these models. We could already use the unsupervised pretrained, so-called base models and present them with sequences such as “I am [MASK]” and “I am not [MASK]” to compare the probabilities that the models assign to informative tokens in place of [MASK]. For diagnostic purposes, we could choose a list of polar adjective pairs (Mathew et al., 2020; Osgood, 1971; Osgood et al., 1957), such as “careful-careless,” that map to psychometric categories of interest (e.g., conscientiousness). The resulting set of probabilities (one per adjective) could then be aggregated to psychological trait scores.

#### Next-word prediction

Another way to train large language models that is used, for example, by the GPT architectures, is trying to predict the next word after a sequence. Similar to the setup using MLM, we could present a model that was trained that way with the sequence “I am” and “I am not” and ask it to output the probability of choosing certain trait adjectives as the next tokens. Those model types are called generative because they are able to create free-form text sequences. This feature could also be directly used in psychometric questionnaires that allow for free-text answering. The scoring of free text is costly, however, as it involves more degrees of freedom and the need for evaluation frameworks (e.g., dictionaries containing trait-related words) to systematically translate answers to numerical scores. The ability of generative models to continue to produce arbitrary sequences of text after they were provided with some initial text creates several problems. First, the initial text with which the model is presented, the so-called prompt, has to be carefully chosen. A whole new discipline of prompt engineering has sprung from the challenge to prompt GPT-type models, as even small variations in the prompts can have substantial and often hard-to-predict effects (P. Liu et al., 2023) on the output the model generates. Second, even when prompts are carefully crafted, the model can (and is expected to) generate different texts in each model run. This element of stochasticity requires collecting model outputs over many iterations and averaging over them. Stochasticity also implies that the model may produce nonsensical or format-incompatible answers in some runs. Third, the model usually has to be provided with some examples to know what output format it is expected to return. Research has shown that even changing the order in the set of examples can influence model outputs (Lu et al., 2022).

#### Zero-shot classification

Yet another approach is zero-shot classification, which relies on pretrained models that have been fine-tuned on NLI text corpora (R. S. Bowman et al., 2015; Conneau et al., 2018; Nie et al., 2020; Williams et al., 2018). These corpora consist of pairs of statements that are labeled according to whether they show entailment, contradiction, or neutrality between the first and the second statement. Learning logical relations of textual entailment between statements in this manner enables models to produce meaningful, often surprising results in other, seemingly disparate domains to which these models were never explicitly introduced. The name “zero-shot” emphasizes the contrast with other approaches that are usually called “few-shot” classification, in which the model is presented with a number of labeled examples for guidance. Different from few-shot classification, models fine-tuned on NLI corpora are able to classify sequences into categories chosen on the fly or to summarize arbitrary texts (Yin et al., 2019) without the need to be presented with any labeled examples before. This feature of domain agnosticism is the hallmark of NLI approaches.

For our demonstrations, we apply such a zero-shot learning approach to elicit model responses to psychometric questionnaires. Our approach combines flexibility with straightforward evaluation and interpretability as an example to illustrate how psychometric assessments of large language models could be implemented. It allows us to prevent certain issues of generative models, in particular, the need for prompt engineering and averaging of stochastic outputs from repeated model runs, although we acknowledge many other ways in which one could set up such demonstrations. We adapt the NLI scheme to present neural models of text with questionnaire items (i.e., statements or questions) from standard psychometric inventories and a corresponding set of verbal response options (e.g., a five-point scale indicating levels of agreement). For each of the response options on the response scale, we record the probability of entailment that the model assigns based on the item wording. We use argmax on these probabilities to assign the most likely response as a score to each item, which we then aggregate into scales using standard scoring procedures, such as taking the sum or mean across all responses pertaining to a (sub)-scale. Models trained on natural language inference (NLI) tasks have been adapted to similar, related contexts before, for example, for questions that have a yes-or-no answer (Clark et al., 2019). Remarkably, for yes–no question answering, an NLI approach shows better performance than a supposedly more direct transfer from multiple-choice answering tasks. In our setup, each of the response options specified by the inventory is a possible answer for the models.

### Example of our approach

To elicit responses to the questionnaire items from the models, we use the procedure visualized in Figure 1: We select the most probable response from the model’s distribution of scores over all responses for each item. To give a concrete example: For the 44-item Big Five Inventory (BFI), item numbers refer to (1) “I am someone who is talkative,” (2) “I am someone who tends to find fault with others,” (3) “I am someone who does a thorough job,” and (4) “I am someone who is depressed, blue,” and so on in English. They translate as (1) “Ich bin gesprächig, unterhalte mich gern;” (2) “Ich neige dazu, andere zu kritisieren”; (3) “Ich erledige Aufgaben gründlich”; and (4) “Ich bin deprimiert, niedergeschlagen,” and so on in German. The response options follow a classic, fully labeled Likert-type scale in which each response category is associated with a specific numerical score. The numerical scores and verbal labels are the following: 1 = *disagree strongly*, 2 = *disagree a little*, 3 = *neither agree nor disagree*, 4 = *agree a little*, and 5 = *agree strongly* in English as well as the equivalent in German. With that, we follow fully the published survey specifications for items, candidate responses, and scoring (John et al., 2008; Rammstedt, 1997).

We want to note here that our approach differs from tests of model performance such as GLUE or SuperGLUE. Such benchmark tests with many subtasks could be viewed as being related to psychological assessments. In the context of natural language understanding, benchmark tests may, for example, include Winograd schemes (Winograd, 1972) and other modules testing the model’s understanding of specific aspects of language. In contrast, we apply psychometric inventories as diagnostic tools that characterize properties of models other than their performance. The distinction between model benchmarks (measuring performance) of models and psychometric inventories (eliciting responses to questionnaire items) is thus reminiscent of the distinction between skills or abilities (e.g., fluid intelligence) and traits (e.g., personality, values) in human psychometrics: In contrast to benchmark tests, when we let models respond to psychometric inventories, there is no ground truth (i.e., “correct” responses to questionnaire items) that a model should display.

Although the goal is thus not to compare the performance of the models as in benchmark tests, the psychometric inventories still allow for a number of meaningful insights into the characteristics of these models. First, we can compare responses of each model to the questionnaire (e.g., the BFI) and its resulting scores on traits such as agreeableness with the distribution of scores (i.e., “norms”) from psychometric assessments in human samples using the same inventories. This allows for relative comparisons of model scores with human averages or typical profiles. For example, a model may be characterized as relatively high in agreeableness if it scores high in that trait relative to typical human populations. Second, independent of absolute scores, the inventories allow us to compare different large language models relative to each other, which in itself is informative about potential differences in the psychological traits these models may exhibit. For example, one model may turn out to score much lower than another in agreeableness. In the future, we might also create reference populations consisting entirely of different models, thereby potentially building a “population of large language models” over time that has a certain distribution of traits (according to highly standardized psychometric inventories) against which future models can be compared.

With our demonstrations, we want to highlight and exemplify the application of various standard questionnaires to large language models. Toward that goal, we recorded the language models’ responses to several psychometric inventories to comprehensively assess the psychological profile of each of these models. These inventories measure constructs from different psychological domains that, although not fully independent, each capture unique aspects of a person’s—or, in our case, a language model’s—psychological profile. Specifically, we chose to assess the global Big Five personality traits, specific “dark” personality traits, value orientations, morality, and gender and sex diversity beliefs. All of these constructs are routinely assessed in research on humans. Moreover, for each construct, inventories that use a fully labeled verbal response scale exist, which was a requirement for our approach. Collectively, these inventories allow us to obtain in-depth psychological profiles of each language model and thereby give us a glimpse into potentially controversial, biased, or harmful characteristics and views that might be ingrained in (some of) these models. They also enable us to compare the psychological profiles of different language models with each other.

For each construct, we chose well-validated inventories that are widely used in research on human. Notably, we do not claim that these are necessarily the best, let alone the only, inventories that would be suitable for the task at hand. We chose these inventories purely for illustrative purposes. In principle, any inventory that works similarly to the ones we chose could be used for psychometric assessment of artificial intelligence. Wherever possible, we use both the English and German version of the same questionnaires to gauge the extent to which results generalize across languages. In the next subsections, we aim to shed light on some of the opportunities that come with deploying psychometric inventories to large language models, using a set of concrete models that are listed in Table A1.

**Table A1.** Different Models Included in Our Demonstrations

Technical name	Short name	Architecture	R.	L.
joeddav/xlm-roberta-large-xnli	XLMRoBERTa	A more robustly pretrained BERT model (RoBERTa) trained using a cross-lingual training objective	Conneau et al., 2020	en, de
cross-encoder/nli-distilroberta-base	DistilRoBERTa	Created using “knowledge distillation” to emulate the output of the larger model (RoBERTa) to create a smaller version	Reimers & Gurevych, 2020	en
microsoft/deberta-base-mnli	DeBERTa	An extension of BERT that introduces new techniques in the model architecture	He et al., 2021	en
MoritzLaurer/mDeBERTa-v3-base-mnli-xnli	multilingual DeBERTa	Fine-tuning DeBERTa on a multilingual natural language inference corpus instead of English only	Laurer et al., 2022	en, de
Sahajtomar/German_Zeroshot	GBERT	BERT pretrained on a corpus of German text finetuned on the German part of XNLI	Chan et al., 2020	de
facebook/bart-large-mnli	BART	Generalizing BERT and GPT training techniques	Lewis et al., 2019	en
valhalla/distilbart-mnli-12-1	DistilBART	Similar procedure as above for DistilRoBERTa but for BART	Shleifer & Rush, 2020	en

Note: Technical name refers to the full model names to be found in the Hugging Face model hub at https://huggingface.co/models. Short name is the abbreviations we use in the visualizations. “R.” describes the citations of model architectures that are briefly summarized under “Architecture.” We used models either in English (en), German (de), or both (“L.”). All of these models have been fine-tuned on one or more of these natural language inference corpora: the Stanford Natural Language Inference corpus (R. S. Bowman et al., 2015), the Multi-Genre NLI corpus (Williams et al., 2018), or XNLI: The Cross-Lingual NLI corpus (Conneau et al., 2018).

## Transparency

*Action Editor:* Melanie Mitchell

*Editor:* Interim Editorial Panel

*Author Contributions*

MP: Conceptualization, Methodology, Formal Analysis, Data Curation, Visualization, Writing - Original Draft; Writing - Review and Editing. CL: Conceptualization, Methodology, Writing - Original Draft, Writing - Review and Editing. CW, BR: Conceptualization, Writing - Review and Editing. MS: Original Conceptualization, Methodology, Writing - Review and Editing. All of the authors approved the final manuscript for submission.

**Open Practices:** We provide a repository that contains materials to fully replicate our analyses at https://github.com/maxpel/psyai_materials.
